# Association Between Body Mass Index and Dental Caries in a Turkish Subpopulation of Adults: A Cross-Sectional Study

**DOI:** 10.3290/j.ohpd.a43935

**Published:** 2020-04-01

**Authors:** Serdar Akarsu, Sultan Aktuğ Karademir

**Affiliations:** a Assistant Professor, Ordu University, Faculty of Dentistry, Department of Restorative Dentistry, Turkey. Wrote and proofread the paper.; b Researcher, Ordu University, Faculty of Dentistry, Department of Restorative Dentistry, Turkey. Research.

**Keywords:** dental caries, DMFT, obesity

## Abstract

**Purpose:**

To evaluate the relationship between decayed, missing and filled teeth (DMFT index) and body mass index (BMI) in a Turkish population of adults aged 20–30 years who did not have any chronic diseases.

**Materials and Methods:**

This study was conducted on a total of 394 patients. DMFT index was used to define the number of teeth with decays, teeth with fillings, and missing teeth. The body weight and height of the study participants were measured with a digital scale and height rod. BMI was calculated by dividing the body weight by the square root of the height. One-way analysis of variance (ANOVA) and Bonferroni tests were used to compare three or more groups and to compare two groups, respectively. The statistical significance level was evaluated at p < 0.05.

**Results:**

Differences in DMFT index among BMI groups was found to be statistically significant (p = 0.001; p < 0.01). DMFT index was significantly higher in the obese group than in the normal-weight group (p = 0.001) and overweight group (p = 0.001). No statistically significant differences were found between DMFT indices of study participants of normal weight and those who were overweight (p > 0.05).

**Conclusion:**

Positive correlation was observed between obesity and DMFT index. Coadministration of obesity prevention programmes and preventive oral health programmes can improve public health to a better point.

Obesity and overweight have been the focus of studies for many years. The prevalence of obesity has increased globally in the last 10 years, and obesity has been recognised as a global pandemic by the World Health Organization. Besides the aesthetic drawbacks, obesity affects many organ systems of the body leading to life-threatening complications. Many factors are considered to be involved in developing obesity including genetic, physical, and environmental factors such as living conditions, dietary habits, and sedentary lifestyles. According to the World Health Organization (WHO), obesity is defined as an extreme or abnormal accumulation of fat which creates a risk to the well-being of the body. Excess weight and obesity, diabetes, cardiovascular diseases and cancer are important risk factors for a number of chronic diseases. Once considered an issue only in high-income countries, obesity and being overweight are currently on the rise in low- and middle-income countries, especially in urban environments.^13^

General health and oral health share similar causalities and behavioural patterns.^23^ Oral health is associated with the general health of the individual.^8^ Excessive consumption of sugar is a well-established common risk factor for both obesity^27^ and tooth decay.^15^ For this reason, it may be suggested that the risk of developing dental caries is high in individuals who are overweight or obese.

WHO has reported that collecting epidemiological data on dental health and diseases is of primary importance.^18^ The most commonly used indices for collecting these data are decayed, missing, filled tooth index (DMFT) and decayed, missing, filled surface index (DMFS). These indices are recommended by WHO to determine and compare the rates of dental caries in a given population.^2^

DMFT index collects data on dental caries, missing teeth, and filled teeth. For individuals under 30 years of age, calculation of DMFT index includes the number of teeth coded as ‘missing teeth, due to caries’. For individuals 30 years of age and older, calculation of DMFT index includes the number of teeth coded as both ‘missing, due to caries’ and ‘missing, due to other reasons’. The total number of teeth used in the calculation of DMFT is a 32, which includes all permanent teeth. The number of teeth coded as being applied a ‘fissure sealant’ or ‘abutment, special crowns or veneers/implants’ is not included in the calculation of DMFT.^12^

Although there is an acceptable relationship between dental caries and obesity, different results have been reported. This study aimed to evaluate the relationship between DMFT index and BMI in a Turkish population of adults aged 20–30 years who did not have any chronic diseases.

## Materials and Methods

This cross-sectional study included 471 volunteers aged 20–30 years who applied to the Clinic of The Department of Restorative Dental Treatment at Ordu University, Faculty of Dentistry between January 2018 and July 2018. All participants signed the informed consent forms, and all procedures in the study were conducted in accordance with the principles of the Helsinki Declaration. This study was approved by the Ethics Committee of Ordu University (2019-31). A total 77 individuals were excluded from the study if they had systemic diseases including diabetes or cardiovascular diseases, were treated for orthodontic treatments, or were pregnant. The sample consisted of 394 individuals.

The study participants were visually examined by an experienced dentist in field restorative dentistry and by means of bite-wing radiography. The DMFT index was used to define the number of teeth with decays, teeth with fillings, and missing teeth.

The body weight and height of the study participants were measured with a digital scale (SECA 888 Digital Scale, Germany) and height rod, respectively. During the measurements, participants were allowed to take off their shoes and measure with thin clothing. While the height measurements were made, the values were recorded as 0.1 cm nearest to the measured value and while the weight measurements were made, the values were recorded as 0.1 kg was the nearest to the measured value. BMI was calculated by dividing the body weight by the square root of the height. The individuals were assigned to the obesity group if their BMI was over 30, to the overweight group if their BMI was within the range of 25–29.9, and to the normal-weight group if their BMI was below 25.

The Number Cruncher Statistical System (NCSS) 2007 (Kaysville, UT, USA) software was used for the statistical analyses of the data. Descriptive statistical methods (mean, standard deviation, median, frequency, ratio, minimum, maximum) were used to assess the data. To compare data conforming to a normal distribution, one-way ANOVA and Bonferroni tests were used to compare three or more groups and to compare two groups, respectively. The level of statistical significance was evaluated at a minimum of p < 0.05.

## Results

**Fig 1:**
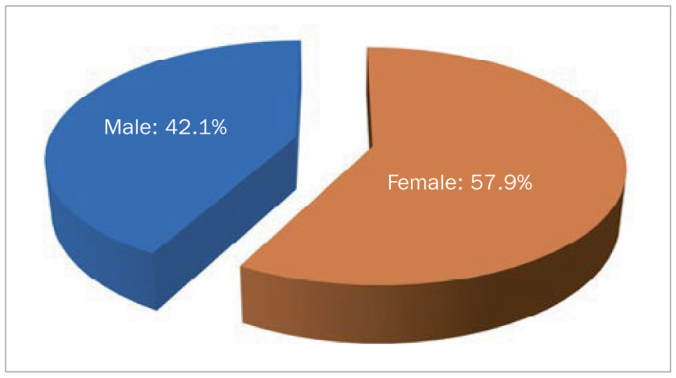
Distribution of sex.

**Fig 2:**
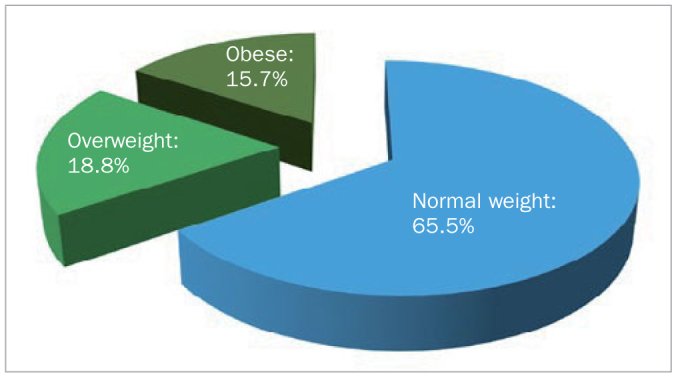
Distribution of BMI.

This study was conducted on a total of 394 patients. Of the total study patients, 57.9% (n = 228) were females and 42.1% (n = 166) were males (Fig 1). When assessing BMI, it was observed that 65.5% (n = 258) of the individuals had normal weight, 18.8% (n = 74) of the individuals were overweight, and 15.7% (n = 62) of the individuals were obese (Fig 2).

All participants were either high-school graduates or university graduates. While they report daily tooth brushing, 14.9% of the participants (40 participants in normal body weight group; 11 participants in overweight group; 8 participants in obese group) brush their teeth two or more times a day.

The number of decayed teeth ranged from 0 to 13 with a mean of 3.02 ± 2.33. The number of extracted teeth ranged from 0 to 8 with a mean of 0.93 ± 1.40. The number of filled teeth ranged from 0 to 17 with a mean of 1.99 ± 2.60. The total number of decayed teeth, extracted teeth, and filled teeth in each individual ranged from 0 to 26, with a mean of 5.94 ± 3.96 (Table 1).

**Table 1:** The scores of D, M, F, DMFT

DMFt		
D (Decayed)	Min–Max (Median)	0–13 (2)
	Mean ± SD	3.02 ± 2.33
M (Missing)	Min–Max (Median)	0–8 (0)
	Meant ± SD	0.93 ± 1.40
F (Filled)	Min–Max (Median)	0–17 (1)
	Mean ± SD	1.99 ± 2.60
DMFt (Decayed + Missing + Filled)	Min–Max (Median)	0–26 (5.5)
	Mean ± SD	5.94 ± 3.96

In females, the DMFT index showed a statistically significant difference among the BMI levels (p = 0.001; p < 0.01). The comparisons of two groups of data to identify the groups, from which the statistically significant difference originated, revealed that the DMFT index of obese subjects was found to be higher (p < 0.01) than either group which had a normal BMI (p = 0.001) and which were overweight (p = 0.001). In females, there was not a statistically significant difference between the DMFT indices of the normal-weight group and the overweight group (p > 0.05).

In males, the DMFT index showed a statistically significant different among the BMI levels (p = 0.011; p < 0.05). Pairing the data in two groups, to determine in which group the data differed significantly, revealed that DMFT index of the obese group was found out to be higher (p < 0.05) compared to the normal-weight (p = 0.011) and overweight groups (p = 0.014). There was not a statistically significant difference between the DMFT indices of the normal-weight group and the overweight groups in males (p > 0.05).

In all study participants, the DMFT index displayed a statistically significant difference amongst the BMI levels (p = 0.001; p < 0.01). The comparisons of two groups of data to identify the groups, from which the statistically significant difference originated, revealed that the DMFT index of obese subjects was found out to be higher (p < 0.01) than either group which had a normal BMI (p = 0.001) and which were overweight (p = 0.001). The DMFT indices of the normal and overweight groups were not significantly different (p > 0.05) (Table 2).

**Table 2:** Evaluation of DMFt according to BMI

		n Min–Max (Median)	DMF t (Decayed + Missing + Filled)		ap
			Mean ± SD		
Female	Normal weight	141	0–12 (6)	5.30 ± 2.69	0.001**
	Overweight	46	0–10 (5)	4.85 ± 3.03	
	Obese	41	1–26 (9)	9.63 ± 5.72	
Male	Normal weight	117	0–17 (5)	5.33 ± 3.34	0.011*
	Overweight	28	1–15 (5)	5.21 ± 3.47	
	Obese	21	1–23 (11)	9.76 ± 6.20	
Total	Normal weight	258 (65.5%)	0–17 (5)	5.32 ± 3.00	0.001**
	Overweight	74 (18.8%)	0–15 (5)	4.99 ± 3.19	
	Obese	62 (15.7%)	1–26 (9.5)	9.68 ± 5.84	

^a^One-way ANOVA Test; * p < 0.05; **p < 0.01.

## Discussion

Obesity is a global public health issue in the 21st century. The rates of obesity are rising daily in both developed and developing countries. WHO reported an increase of 10–30% in the prevalence of obesity between the years 1980–1990 according to the results of the MONICA (monitoring trends and determinants in cardiovascular disease) study, which was conducted in six different sites in Asia, Africa and Europe for a duration of 12 years.^10^ Between 1980 and 2008, the prevalence of obesity doubled in the world. In 2008, 10% of males and 14% of females became obese (BMI ≥ 30 kg/m^2^), although the rates were 5% for males and 8% for females in 1980. In 2008, approximately 205 million males and 297 million females over the age of 20 became obese, which accounts for more than half a billion adults all over the world.^14,20^ In Australia, one in four children and two in three adults are either obese or overweight^25^ and the data are similar to those collected in our country. The Ministry of Health in Turkey published a paper in 2018 reporting that one in every five individuals was obese.^6^

Although it has been reported in the literature that there is an acceptable biological association between dental caries and obesity, varying reports are available in the literature.^7,16,24^

Individuals with a body weight lower than normal may have a higher risk for having decayed teeth. Narksawat et al conducted a cross-sectional study in children between aged 12 and 14 years and reported that children with a normal or lower than normal weight had higher DMFT indices than children who were overweight and obese.^11^

In another cross-sectional study, Gonçalves et al reported that children and adolescents with higher BMI indices had lower DMFT scores and that individuals who did not consume fruits and vegetables had higher DMFT scores.^5^

Insufficient nutrition leads to a decreased flow rate of saliva and a reduced capacity for buffering. The decrease in the rate of the salivary flow and the reduction in the capacity of buffering are factors leading to the development of dental caries.^19^

One of the potential risk factors in the association between dental caries and obesity is the high rate of consumption of food rich in sugar and fat. It has been observed that being overweight and obese is more frequent in children from high socioeconomic groups. The tendency of children to consume fermented carbohydrates by parents in the high socioeconomic groups suggests that these factors may increase the risk for developing obesity and similarly dental caries as well.^4^

Most of the previous studies on obesity and dental caries were conducted on preschool children and on adolescents. The number of studies evaluating the association between dental caries and obesity on adults is limited. Sakeenabi et al conducted a study to investigate the association between obesity and dental caries and reported a significantly positive correlation between obesity and tooth decay.^22^ Khaled Alswat et al conducted a study on a group of 385 individuals with a mean age of 28.39 years and reported that 55.3% of individuals were obese or overweight and DMTF index was 6.55. They observed a significantly positive correlation between the BMI and DMFT index.^1^ Verma et al conducted a study on 1125 adults aged between 25 and 44 years and reported that 21.2% were either overweight or obese and that DMFT index was 4.39 and 5.53 in the overweight and obese individuals, respectively. They also reported a significantly positive correlation between the BMI and DMFT index.^28^ These data are similar to those of our study. In our study, of the 394 study participants with a mean age of 23.8 years, 15.7% were obese and had a DMFT index of 9.68. The DMFT index of the obese individuals was found to be higher compared to individuals of either normal weight (DMFT index 5.32; p = 0.001) or overweight (DMFT index 4.99; p = 0.001).

A study conducted to investigate the association between the BMI and the level of oral health on adults from Korea could not find a correlation between obesity and dental caries.^7,9^ Since systemic diseases including type 2 diabetes and cardiovascular disorders are more common in individuals in Asia with a lower body weight, the BMI thresholds used in the study differed from those used in our study. In our study, the range of BMI signifying overweight was 25–29.9, and the threshold for obesity was defined as having a BMI of 30 or over. However, in the abovementioned study conducted on individuals from Korea, the ranges were defined as 23–24.9 for the overweight individuals, and the threshold for obesity was defined as having a BMI of 25 and over. Therefore, we are of the opinion that comparing studies using different threshold values for BMI will not be appropriate.

Dental caries is a chronic, highly prevalent and cumulative non-communicable disease which develops over time.^17^ Oral health behaviours are essential for oral health. Some studies suggest that tooth brushing play an important role in reducing dental caries and periodontal disease.^3^ Frequency of tooth brushing during their childhood and adolescence can affect prevalence of dental caries in adulthood. Although all participants tell them frequency of tooth brushing every day in this study, they could not give precise information about the frequency of tooth brushing during their childhood and adolescence.

The BMI is a beneficial measure to define being overweight and obese both in males and females. It is easy to calculate. However, the concept of BMI has statistically significant limitations. It neglects several factors including the differences in the muscle mass, bone mass and genetic features. Therefore, the BMI may not be an appropriate measure for overweight and obese individuals when used alone. For example, the BMI may provide an extremely high ratio of fat in athletic individuals having an abundant muscle mass.^21^ Therefore, measuring the waist circumference and evaluation of this measure in combination with the BMI values may provide more accurate results.

All participants were living in Ordu in Turkey. The United States Public Health Service now recommends an optimal fluoride concentration of 0.7 mg/L.^26^ The optimal concentration of fluoride in drinking water is the concentration that provides the best balance of protection from dental caries while limiting the risk of dental fluorosis. The fluoride concentration of drinking water in Ordu is low (0.061 mg/L). The addition of fluoride to drinking water is not practised in Turkey.

Our study has concluded that DMFT index is higher in obese individuals. However, our study has some limitations. Although our study sample is representative of a defined population, it was conducted only in one country. Multinational studies with the participation of different genetic groups are required to ensure the validity of the studies. In addition, it was not possible to assess causality in our study because we collected cross-sectional data. More extensive and prospective studies are required to determine the causal relationship between dental caries and obesity in order to intervene in the variables examined in the study.^24,29^

## Conclusion

According to our study results, we can say that there is a positive relationship between obesity and tooth decay. Therefore, coadministration of obesity prevention programmes and preventive oral health programmes can improve public health to a better level.
